# Sociocultural variability in the Latino population: Age patterns and differences in morbidity among older US adults

**DOI:** 10.4054/DemRes.2018.38.52

**Published:** 2018-05-15

**Authors:** Catherine Garcia, Marc A. Garcia, Jennifer A. Ailshire

**Affiliations:** 1 University of Southern California, Los Angeles, USA; 2 Sealy Center on Aging, University of Texas Medical Branch, Galveston, USA; 3 University of Southern California, Los Angeles, USA

## Abstract

**BACKGROUND:**

The US Latino population is rapidly aging and becoming increasingly diverse with respect to nativity and national origin. Increased longevity along with medical advancements in treatment have resulted in a higher number of older Latinos living with morbidity. Therefore, there is a need to understand variability in Latino health among older adults.

**OBJECTIVES:**

This paper documents mid- and late-life health differences in morbidity by race/ethnicity, nativity, and country of origin among adults aged 50 and older.

**METHODS:**

We use data from the 2000–2015 National Health Interview Survey to calculate age-and gender-specific proportions based on reports of five morbidity measures: hypertension, heart disease, stroke, cancer, and diabetes among non-Latino Whites and seven Latino subgroups.

**RESULTS:**

The foreign-born from Mexico, Cuba, and Central/South America, regardless of gender, exhibit an immigrant advantage for heart disease and cancer in comparison to non-Latino Whites across all age categories. Conversely, island-born Puerto Ricans are generally characterized with higher levels of morbidity. Similarly, US-born Puerto Ricans and Mexicans exhibit morbidity patterns indicative of their minority status. Latinos, regardless of gender, were more likely to report diabetes than non-Latino Whites. Hypertension and stroke have significant variability in age patterns among US-and foreign-born Latinos.

**CONCLUSION:**

Recognizing the importance of within-Latino heterogeneity in health is imperative if researchers are to implement social services and health policies aimed at ameliorating the risk of disease.

**CONTRIBUTION:**

Considering intersectional ethnic, nativity, and country-of-origin characteristics among older Latinos is important to better understand the underlying causes of racial/ethnic disparities in morbidity across the life course.

## 1. Introduction

The older US population has experienced unprecedented growth, with aging Latinos constituting a significant part of this increase (West et al. 2014). Despite their low socioeconomic status, older Latinos, particularly the foreign-born, have longer life expectancies and lower mortality than non-Latino Whites (Fenelon, Chinn, and Anderson 2017; Garcia et al. 2017). The combination of a poor socioeconomic profile with increased longevity may present key challenges for the health of older Latinos. For instance, decreases in mortality without subsequent improvements in morbidity leads to a higher proportion of the population living with diabetes, hypertension, heart disease, and other chronic illnesses.

However, Latinos are not a monolithic group, and their health in later life may vary depending on sociocultural factors such as nativity and country of origin. Research shows nativity status to be a crucial factor for determining individual health. In general, US-born Latinos have a health profile more indicative of their minority status, exhibiting higher rates of morbidity (Garcia and Reyes 2017; Zhang, Hayward, and Lu 2012) and spending more of their later-life years with a chronic condition than foreign-born Latinos (Cantu et al. 2013; Garcia et al. 2017).

Migration selectivity, which is strongest among foreign-born Mexicans (Palloni and Arias 2004), is thought to shape the health profiles of Latino immigrants (Bostean 2013). Selective migration is defined as disproportionate migration to the United States by individuals in good health compared to those in poor health (Riosmena, Wong, and Palloni 2013). However, research examining health selectivity among older Latinos from the Caribbean and Latin America is scarce, particularly in regard to morbidity. This is an important factor considering the range of immigration experiences (i.e., as influenced by country of origin, sociopolitical status, socioeconomic position, and gender).

For instance, older Puerto Rican and foreign-born Cuban adults experience different sociopolitical circumstances related to migration relative to other foreign-born Latino groups. Island-born Puerto Ricans are natural-born US citizens, thus allowing them to migrate to the US mainland more easily than other Latino subgroups (Duany 2011). Internal migration from Puerto Rico to the US mainland distinctly differs from international migration from Mexico, the Caribbean, or other Latin America countries; as such, migration selectivity may play less of a role for island-born Puerto Ricans. Conversely, many foreign-born Cubans entered the United States as political refugees and were granted a pathway toward citizenship along with social and economic services (Saenz and Morales 2015) that may be protective of late-life health.

Recent research suggests substantial variability in morbidity among Latino subgroups by nativity and country of origin relative to non-Latino Whites. However, the extant literature exploring morbidity among Latinos by nativity and country of origin has largely been limited to regional studies and has not focused specifically on older adults (Lin et al. 2002; Sacco et al. 2001; Borrell et al. 2011; Zsembik and Fennell 2005). Thus, this paper builds on previous research by providing a comprehensive analysis of five measures of morbidity for seven Latino subgroups, stratified by gender and three age categories that encompass mid- and late-life stages of the life course.

## 2. Data and methods

We used data from the National Health Interview Survey (NHIS), an annual household survey of the noninstitutionalized civilian population to estimate morbidity differences among adults aged 50 and older (Parsons et al. 2014). Data is pooled across 16 years (2000–2015) to obtain a large sample of Latino subgroups by nativity and country of origin. The annual response rate of the NHIS is approximately 80%. After excluding respondents with missing data on health (n = 615), our final analytical sample was 164,616.

### 2.1 Measures

We focus on five self-reported morbidity measures: hypertension, heart disease, diabetes, cancer, and stroke, which are among the leading causes of disability and mortality in the United States (Johnson et al. 2014; Manini 2011). Respondents were asked, “Has a doctor ever told you that you have (had a) [condition]?” Responses were coded: 1 = yes and 0 = no.

Sociodemographic variables include race/ethnicity, nativity, gender, and age. In the NHIS, respondents were asked to identify their race, regardless of ethnic identification. For the purpose of this study, we classify Latinos as individuals who self-identify as Latino-origin regardless of racial identification. Respondents were categorized as US-born if they were born in one of the 50 states and foreign-born if they were born outside the United States, including its territories. Self-reported race/ethnicity, birthplace, and country of origin were used to classify respondents. They include US-born Mexicans, foreign-born Mexicans, US-born Puerto Ricans, island-born Puerto Ricans, foreign-born Cubans, foreign-born Dominicans, foreign-born Central/South Americans, and US-born non-Latino Whites. US-born Cubans, Dominicans, and Central/South Americans are excluded due to small sample sizes. We include three age categories that allow for reasonable sample sizes and encompass distinct stages of life – 50–59, 60–69, and 70+ – to assess how morbidity patterns vary by age.

### 2.2 Analytic strategy

We document the proportion of individuals with morbidity by race/ethnicity, nativity, country of origin, and age group. We compare differences between non-Latino Whites and Latino subgroups using chi-squared tests. Estimates are done separately for women and men to account for well-known gender differences in morbidity (Vlassoff 2007). Sample weights were used to correct for differential selection and nonresponse bias and include a poststratification adjustment to make the sample representative of the US population. We account for the complex survey design of the NHIS by using Stata’s *svy* commands, which adjust for population stratification, primary sampling unit, and sample weights.

## 3. Results

Table 1 shows the prevalence of five self-reported chronic conditions in three age categories among women. US- and foreign-born Mexicans, island-born Puerto Ricans, and foreign-born Dominicans exhibited significantly higher proportions of hypertension than non-Latina Whites across age groups. Conversely, foreign-born Cubans and foreign-born Central/South Americans exhibited rates of hypertension comparable to those of non-Latina Whites across all age categories, with the exception of foreign-born Cubans in the 60–69 age group. Within Latina subgroups, island-born Puerto Ricans and foreign-born Dominicans have a significantly higher prevalence of hypertension than foreign-born Mexicans across age categories.

In contrast, all Latina subgroups reported comparable or lower levels of heart disease than non-Latina White women across age categories, except for island-born Puerto Rican women, who report a significantly higher prevalence of heart disease at ages 50–59. For stroke, US-born Mexicans (4%) were more likely to report stroke at ages 50–59 than non-Latina Whites (2%). At ages 60–69 US-born Mexican (5%), US-born Puerto Rican (7%), island-born Puerto Rican (8%), and foreign-born Dominican (6%) women had a significantly higher proportion of stroke than non-Latina Whites (4%). This pattern of disadvantage among Latina subgroups converges at ages 70 and older to that of non-Latina Whites. However, foreign-born Cubans exhibited a significantly lower prevalence of stroke at ages 70 and older. In addition, all Latina women, regardless of nativity or country of origin, reported a significantly lower prevalence of cancer relative to non-Latina Whites across age categories, with foreign-born Dominicans exhibiting the lowest proportions among Latina subgroups.

In general, all Latina subgroups (with the exception of foreign-born Cubans) reported a significant disadvantage in the prevalence of diabetes relative to non-Latina Whites. However, there was significant variability within Latina subgroups. For instance, US- and foreign-born Mexicans and island-born Puerto Ricans were twice as likely to report diabetes than non-Latina Whites across age categories. Among Latina women, US-born Puerto Rican (13%), foreign-born Cuban (11%), foreign-born Dominican (12%), and foreign-born Central/South American (12%) women were significantly less likely to report diabetes than foreign-born Mexicans (19%) at ages 50–59. These patterns largely remain consistent across age categories, though disparities between foreign-born Mexicans and foreign-born Dominicans in diabetes converge at ages 60–69, while disparities between foreign-born Mexicans and US-born Puerto Ricans converge at ages 70 and older.

Table 2 shows the prevalence of chronic conditions among men. Foreign-born Mexicans and foreign-born Central/South Americans exhibited a significantly lower prevalence of hypertension than non-Latino Whites across age groups, except for foreign-born Central/South Americans at ages 70 and older. Conversely, US-born Mexican, island-born Puerto Rican, and foreign-born Dominican men exhibited significantly higher proportions of hypertension than non-Latino Whites at ages 50–59. However, the observed patterns of disadvantage converge at ages 60 and older to those of non-Latino Whites. For heart disease, Latino men exhibited comparable or significantly lower rates compared to non-Latino Whites across age categories. Overall, foreign-born migrants from Mexico, Cuba, and Central/South America exhibited an immigrant advantage (selectivity) in the proportion reporting heart disease relative to US-born Latinos.

For stroke, US-born Mexicans (4%), US-born Puerto Ricans (8%), and island-born Puerto Ricans (4%) exhibit a significantly higher proportion of stroke than non-Latino Whites (3%) at ages 50–59, whereas foreign-born Central/South Americans were significantly less likely to report stroke (1%). At ages 60–69, US-born Mexicans and island-born Puerto Ricans continue to exhibit a higher prevalence of stroke than non-Latino Whites, whereas the disadvantage in stroke between US-born Puerto Ricans and non-Latino Whites largely disappears. In late life (70 and older), the disadvantage experienced by US-born Mexicans and island-born Puerto Ricans converges to that of non-Latino Whites. In addition, all Latino men, regardless of nativity or country of origin, report a comparable or significantly lower prevalence of cancer than non-Latino Whites across age categories. Foreign-born Mexicans exhibit the lowest proportions among Latino subgroups.

Finally, all Latino subgroups, with the exception of foreign-born Cubans and foreign-born Central/South Americans, report a significant disadvantage in the prevalence of diabetes relative to non-Latino Whites. However, significant variability exists within Latino subgroups. In general, US-born Mexicans, island-born Puerto Ricans, and, to a lesser extent, foreign-born Dominicans exhibited a higher prevalence of diabetes than other Latino subgroups.

## 4. Discussion

Racial/ethnic, nativity, and country-of-origin differences in morbidity among older adults are of crucial importance because they highlight the consequences of social inequality in US society. These inequalities result in differing health trajectories across population subgroups in late life. The rationale underlying our study is that the Latino population is heterogeneous in nativity and country of origin, which is often overlooked in health research. Mexican-origin individuals make up approximately two-thirds of the Latino population, and as a result, many statistics on the health of Latinos are disproportionately impacted by their numbers.

Our results indicate substantial variation in health among Latinos by morbidity measure, nativity, country of origin, and gender. First, we document comparable or lower prevalence of heart disease and cancer among Latinos relative to non-Latino Whites. Second, we find foreign-born older adults from Mexico, Cuba, and Central/South America, regardless of gender, exhibit a clear immigrant advantage for heart disease and cancer relative to non-Latino Whites across all age categories. Prior studies suggest that socioeconomic differences play an important part in these differences (Hummer, Benjamins, and Rogers 2004). The substantial health advantage of older foreign-born Cuban and Central/South Americans is likely due to their more favorable socioeconomic status relative to other Latino groups. For example, scholars have documented the creation of Cuban ethnic enclaves that provide economic opportunity structures that promote upward mobility (Portes and Stepick 1994). In addition, Central/South Americans are more likely to be employed than other Latino subgroups, which contributes to their higher earnings (Lesser and Batalova 2017; Zong and Batalova 2016). The socioeconomic advantages enjoyed by Cubans and Central/South Americans relative to other Latino subgroups likely translate to better health profiles across the life course through increased access to goods, services, and health care. In contrast, immigrants from Mexico are more likely to be selected on health despite their low socioeconomic status. Evidence shows Mexican migrants have a lower prevalence of chronic conditions relative to nonmigrants, which provides support for migration selection (Bostean 2013; Riosmena, Wong, and Palloni 2013).

Third, island-born Puerto Ricans are generally characterized by higher levels of morbidity than non-Latino Whites. Island-born Puerto Ricans are US citizens and can travel freely to the US mainland without a visa, making them less selected for health as they do not have to deal with obstacles related to the migration process encountered by other migrants (Duany 2011). Similarly, US-born Puerto Ricans and US-born Mexicans exhibit morbidity patterns indicative of their minority status. Previous research shows Puerto Ricans in the United States live in segregated neighborhoods characterized by social and economic isolation, which is linked to an increased risk of poor health. Santiago and Galster (1995) provide a thorough analysis of the deteriorating economic status of Puerto Ricans, which selects them into segregated neighborhoods with inadequate access to health care facilities, limited options for nutritious food, lack of recreational activities, and the absence of generational socioeconomic upward mobility. Conversely, US-born Mexicans may be at greater risk for morbidity due to experiencing greater stress related to their low socioeconomic status (Gallo et al. 2013). Among Mexican American women, financial stress has been found to be associated with elevated levels of allostatic load – an indicator of physiological dysregulation across multiple systems that predicts greater morbidity (Gallo et al. 2011).

Fourth, Latinos, regardless of gender, were more likely to report diabetes than non-Latino Whites. However, we document significant variation in the prevalence of diabetes. Foreign-born Cuban women and foreign-born Central/South American men were less likely to report diabetes than their non-Latino White counterparts. In contrast, US-born Mexican, foreign-born Mexican, and island-born Puerto Rican men and women had a higher proportion of diabetes relative to other groups. Cardiometabolic abnormalities, which are associated with an elevated risk of diabetes, are high among Latinos, particularly individuals of Puerto Rican descent (Heiss et al. 2014). Finally, we document a higher prevalence of hypertension among Latina women relative to non-Latina White women, a pattern largely absent among Latino men. It has been found that for individuals aged 65 or older, hypertension affects more women than men (Mozzafarian et al. 2015), which may be attributed to women’s more disadvantaged social and economic positions throughout the life course (which accumulate toward late life) relative to men. Moreover, hypertension tends to be a comorbid condition associated with diabetes and increases the risk for cardiovascular and cerebrovascular disease. With the higher prevalence of diabetes and hypertension among Latina women, it is not surprising that they have elevated levels of stroke in midlife since diabetes and hypertension are established risk factors for stroke.

This study has several limitations. First, we pooled data over 16 years to obtain a large sample of Latino subgroups by nativity and country of origin. The benefit of a pooled sample lies in the increase of the reliability and precision of estimates that would be difficult to achieve otherwise using cross-sectional data with small cell sizes. However, in pooling data we make the assumption that population differences are stable across survey years. Although socioeconomic, cultural, and health profiles may vary across cohorts, the amount of variation is likely small over the study period. Second, recent evidence suggests third-generation adults are less likely to identify as Latino compared to their first- and second-generation counterparts (Lopez, Gonzalez-Barrera, and Lopez 2017). The likelihood of a person’s identifying as Latino in subsequent generations is also reduced with higher levels of education, ethnic intermarriage, and living in non-Latino neighborhoods (Antman, Duncan, and Trejo 2016; Duncan and Trejo 2011; Lopez, Gonzalez-Barrera, and Lopez 2017). These factors can lead to a downward bias in estimates of health for US-born Latinos, which may make them appear in worse health than they actually are. Third, the use of the Central/South American ethnic grouping is problematic given that two different and diverse regions of Latin America are combined. The Central/South American region encompasses over 20 countries that represent 25% of the total Latino population in the United States. The NHIS does not provide detailed information on Latinos from Central/South America. Nevertheless, we included this category to demonstrate the heterogeneity within the Latino population. Finally, data for this study is obtained from self-reports of doctor-diagnosed conditions, which may introduce bias due to differential reporting between groups. Research suggests Latinos are less likely to be diagnosed with chronic conditions due to lower rates of health insurance coverage, reduced health care access, differences in utilization/source of care, and language/cultural barriers (Tienda and Mitchell 2006).

Despite these limitations, the current study represents an important contribution to knowledge on the complex patterns of older adult health. By including a broad range of Latino subgroups, we document patterns of morbidity that are less apparent in research that focuses on Latinos as a homogenous group. Recognizing the various social processes that underlie Latino populations is important if researchers are to fully understand the health of minority and immigrant groups.

## Figures and Tables

**Table 1:** Proportion reporting a chronic condition, by race/ethnicity/nativity among females by age group, 2000–2015

	Hypertension			Heart disease			Stroke			Cancer			Diabetes		
	50–59	60–69	70+	50–59	60–69	70+	50–59	60–69	70+	50–59	60–69	70+	50–59	60–69	70+
US Mexican	0.38^a^	0.57^a^	0.64^a^	0.10^a^	0.16	0.22^a^	0.04^a^	0.05^a^	0.10	0.07^a^	0.12^a^^b^	0.12^a^^b^	0.19^a^	0.29^a^^b^	0.33^a^
FB Mexican	0.37^a^	0.60^a^	0.67^a^	0.08^a^	0.14^a^	0.22^a^	0.03	0.04	0.07	0.05^a^	0.05^a^	0.09^a^	0.20^a^	0.31^a^	0.33^a^
US Puerto Rican	0.39^a^	0.50	0.66	0.15^b^	0.10	0.23	0.04	0.07^a^^b^	0.17^b^	0.09^b^	0.11^a^	0.12^a^	0.13^a^^b^	0.21^a^^b^	0.26
IB Puerto Rican	0.47^a^^b^	0.64^a^^b^	0.68^a^^b^	0.15^a^^b^	0.18^b^	0.27^b^	0.03	0.08^a^^b^	0.10	0.09^a^^b^	0.11^a^^b^	0.11^a^	0.25^a^	0.30^a^	0.29^a^
FB Cuban	0.33	0.55^a^	0.66	0.06^a^	0.09^a^^b^	0.18^a^	0.02	0.04	0.07^a^	0.04^a^	0.06^a^	0.11^a^	0.11^b^	0.14^b^	0.18^a^^b^
FB Dominican	0.45^a^^b^	0.68^a^^b^	0.71^a^^b^	0.09	0.15	0.17^a^	0.02	0.06^a^^b^	0.10	0.01^a^^b^	0.05^a^	0.08^a^	0.12^a^^b^	0.29^a^	0.24^a^
FB Central/South American	0.33^b^	0.46^b^	0.59^b^	0.05^a^	0.10^a^^b^	0.19^a^	0.01^b^	0.03	0.08	0.06^a^	0.07^a^^b^	0.08^a^	0.12^a^^b^	0.17^a^^b^	0.21^a^^b^
US White	0.33^b^	0.48^b^	0.62^b^	0.12^b^	0.17^b^	0.29^b^	0.02	0.04	0.09	0.12^b^	0.18^b^	0.24^b^	0.09^b^	0.13^b^	0.14^b^

*Note*: US = US-born; FB = foreign-born; IB = island-born.

a indicates that the proportion differs from U.S.-born Whites (*p*<.05).

b indicates that the proportion differs from foreign-born Mexicans (*p*<.05).

**Table 2:** Proportion reporting a chronic condition, by race/ethnicity/nativity among males by age group, 2000–2015

	Hypertension			Heart disease			Stroke			Cancer			Diabetes		
	50–59	60–69	70+	50–59	60–69	70+	50–59	60–69	70+	50–59	60–69	70+	50–59	60–69	70+
US Mexican	0.43^a^^b^	0.56^b^	0.59^b^	0.12^b^	0.21^a^^b^	0.28^a^	0.04^a^^b^	0.09^a^^b^	0.10	0.03^a^^b^	0.10^a^^b^	0.15^a^^b^	0.21^a^	0.32^a^^b^	0.34^a^^b^
FB Mexican	0.29^a^	0.45^a^	0.51^a^	0.06^a^	0.17^a^	0.27^a^	0.02	0.05	0.09	0.02^a^	0.04^a^	0.11^a^	0.19^a^	0.26^a^	0.27^a^
US Puerto Rican	0.37^b^	0.49	0.64	0.16^b^	0.13	0.48^b^	0.08^a^^b^	0.01	0.10	0.09^b^	0.13^b^	0.37^b^	0.16^a^	0.28^a^	0.37^a^
IB Puerto Rican	0.46^a^^b^	0.51^b^	0.60	0.14^b^	0.24^b^	0.27^a^	0.04^a^^b^	0.09^a^^b^	0.12	0.02^a^	0.09^a^^b^	0.14^a^	0.22^a^	0.35^a^^b^	0.36^a^
FB Cuban	0.36^b^	0.49	0.51^a^	0.09^a^	0.19^a^	0.27^a^	0.01	0.03	0.08^a^^b^	0.04^a^^b^	0.10^a^^b^	0.22^a^^b^	0.13^b^	0.16^b^	0.23
FB Dominican	0.49^a^^b^	0.62^b^	0.60	0.10	0.17	0.33	0.02	0.06	0.17^b^	0.02^a^	0.09^b^	0.11^a^	0.21^a^	0.20	0.43^a^^b^
FB Central/South American	0.24^a^^b^	0.42^a^	0.52	0.05	0.16^a^	0.21^a^	0.01^a^^b^	0.03	0.08	0.02^a^	0.07^a^	0.13^a^	0.11^b^	0.20	0.22
US White	0.38^b^	0.52^b^	0.57^b^	0.14^b^	0.26^b^	0.41^b^	0.03	0.05	0.10	0.08^b^	0.18^b^	0.31^b^	0.10^b^	0.17^b^	0.19^b^

Note: US = US-born; FB = foreign-born; IB = island-born.

a indicates that the proportion differs from US-born Whites (*p*<.05).

b indicates that the proportion differs from foreign-born Mexicans (*p*<.05).
